# Lava Mapping Using Sentinel-1 Data after the Occurrence of a Volcanic Eruption—The Case of Cumbre Vieja Eruption on La Palma, Canary Islands, Spain

**DOI:** 10.3390/s22228768

**Published:** 2022-11-13

**Authors:** Aggeliki Kyriou, Konstantinos G. Nikolakopoulos

**Affiliations:** Department of Geology, University of Patras, 265 04 Patras, Greece

**Keywords:** lava mapping, volcanic eruption, Sentinel-1, interferometry, offset tracking

## Abstract

Volcanic eruptions pose a great threat to humans. In this context, volcanic hazard and risk assessment constitute crucial issues with respect to mitigating the effects of volcanic activity and ensuring the health and safety of inhabitants. Lava flows directly affect communities living near active volcanoes. Nowadays, remote sensing advances make it possible to effectively monitor eruptive activity, providing immediate and accurate information concerning lava evolution. The current research focuses on the mapping of the surface deformation and the analysis of lava flow evolution occurred on the island of La Palma, during the recent (2021) eruptive phase of the volcano. Sentinel-1 data covering the island were collected throughout the entire eruptive period, i.e., September 2021 until January 2022. The processing was based on amplitude-based and phase-based detection methods, i.e., Synthetic Aperture Radar interferometry (InSAR) and offset tracking. In particular, ground deformation occurred on the island, while Line-Of-Sight (LOS) displacements were derived from Sentinel-1 interferograms. Moreover, the evolution of lava flow velocity was estimated using Sentinel-1 imagery along with offset tracking technique. The maximum lava flow velocity was calculated to be 2 m/day. It was proved that both approaches can provide rapid and useful information in emergencies, especially in inaccessible areas. Although offset tracking seems a quite promising technique for the mapping of lava flows, it still requires improvement.

## 1. Introduction

Volcanic eruptions are one of the greatest threats to humans. According to the World Health Organization, millions of people were affected and thousands lost their lives from volcanic activities between 1998 and 2017 [1]. In addition, the spewing of hot/dangerous gases, ash, lava and rocks can cause catastrophic losses to essential services (water, transport, communications, etc.), infrastructure and the environment. There are approximately 1500 active or potentially active volcanoes worldwide, while a significant percentage of the population lives in their vicinity. Therefore, volcanic hazard and risk assessment are crucial issues with respect to mitigating the effects of the volcanic activity and ensuring the health and safety of inhabitants. Lava flows are a serious hazard to communities living close to active volcanoes, as the destruction they cause is commonly large in scale [2,3]. In this context, local communities need immediate and accurate information regarding the spread of newly erupted lava [4].

Advances in remote sensing technology have opened up new opportunities in the monitoring of eruptive activity, offering a vast amount of data with a wide range of temporal and spatial resolution, acquired using different sensors [5,6,7,8,9]. The rapid monitoring and assessment of volcanic hazard over large-scale and/or inaccessible areas are the main advantages of such approaches, contributing to a more comprehensive understanding of ongoing volcanic processes. Synthetic Aperture Radar (SAR) has emerged as a valuable source of information for the monitoring and assessment of volcanic hazard, since this specific type of sensor is able to collect data throughout the day under any atmospheric conditions [10,11,12]. In the first attempts, SAR imagery was processed along with multispectral (Landsat, ASTER, SPOT, etc.) or topographical (Shuttle Radar Topography Mission/ASTER DEM) data, aiming at the mapping, quantification and investigation of the lava flows in terms of structure, thickness, morphology and texture [13,14]. Moreover, coherence-based SAR techniques have proven particularly effective in detecting terrain changes and active flow paths, arising from lava flows, as radar signal is directly affected by the modifications of surface’s scattering properties [15,16].

Interferometric Synthetic Aperture Radar (InSAR) is one of the most effective and widely applied satellite techniques for the precise measurement of the Earth’s deformation. Thus, SAR interferograms created by TerraSAR-X, Envisat, ERS, COSMO-SkyMed and ALOS data have been exploited as operational tools for the detection and analysis of surface deformation, which is related to volcanic activity, as well as for the determination of long-term lava subsidence and behavior [17,18,19]. The launch of the Sentinel-1 constellation dramatically increased the amount of SAR imagery, allowing scientists to take a look at volcanoes’ inner processes and eruption mechanisms [20]. Meanwhile, advanced interferometric techniques such as Persistent Scatterer Interferometry (PSI) and Multi-Temporal InSAR (MT-InSAR) have been developed in order to overcome the limitations (i.e., temporal decorrelation, atmospheric contribution) of the conventional interferometric approaches and to assess volcanic deformation in a more reliable way [21,22]. In this context, the Committee on Earth Observation Satellites (CEOS) undertook a four-year (2013–2017) pilot project for the cost-effective and efficient monitoring of volcanoes around the world, using multi-satellite radar imagery along with interferometric techniques [23]. The most recent studies report the synergistic use of multi-sensor remote sensing data (radar, multispectral, etc.) with the aim of effectively mapping lava flows and achieving real-time assessment of volcanic hazards. These automated approaches are based on hierarchical split-based methods, machine learning algorithms, or convolutional neural networks [24,25,26].

The current research focuses on the mapping of surface deformation and the analysis of the evolution of lava flows occurring on the island of La Palma during the recent eruptive phase of the volcano. To this end, Sentinel-1 data over the island were collected, covering the entire eruptive period, i.e., September 2021 until January 2022. The processing was based on the exploitation of both amplitude and phase information derived from SAR’s backscattering signal. Specifically, Sentinel-1 interferograms were created throughout the investigated period and ground deformation was estimated using Line-Of-Sight (LOS) displacements. Moreover, the offset tracking technique constitutes a powerful approach for the mapping of displacements in both azimuth and slant-range direction. The method applies a cross-correlation algorithm between a SAR reference and secondary images, and it is based on feature similarity of homologous intensity patches. Therefore, feature velocities of the intensity patches are calculated. This technique has been widely used in the investigation of glacier motion [27,28,29], and has recently been used in the identification of terrain changes related to landslide phenomena [30,31]. In volcanic research, offset tracking has been applied in a few studies associated with either the growth of the lava dome or the detection of instabilities on the flanks of a volcano [32,33]. The current study suggests that the offset tracking technique can also be used in the mapping of lava flows as they proceed from the crater to their endpoint. Hence, this is the main contribution of the current research.

In summary, this work proposes an almost near-real-time monitoring approach for the analysis of the surface deformation and lava flow evolution during a volcanic crisis. Ground displacements as well as lava flow velocities are the main processing outcomes, and can be used effectively for the proper design and implementation of holistic risk management plans. Specifically, awareness of lava flow velocity is a crucial parameter for local stakeholders in order to safely evacuate nearby settlements, reducing the potential risk of human casualties. Characteristic examples of how catastrophic a volcanic eruption could be include the Mont Pelee and Krakatau eruptions. More specifically, pyroclastic flow caused by the 1902 eruption of Mont Pelee, in Martinique, Lesser Antilles, destroyed the coastal city of St. Pierre, killing about 30,000 inhabitants. In addition, 36,000 people lost their lives in the Krakatau volcanic eruption in 1883.

In recent years, the evolution of remote sensing technology has contributed to the minimization of human casualties; however, volcanic eruptions still result in extreme damage to the environment and infrastructure. SAR technology offers almost-real-time updates for lava flow direction and velocity that could be used in synergy with relief information to perform the immediate calculation of the distance between the lava and the nearest settlements and provide an accurate estimation of the remaining time available for evacuation.

## 2. Materials and Methods

### 2.1. Area of Interest—La Palma Volcanic Eruption

**Figure 1:**
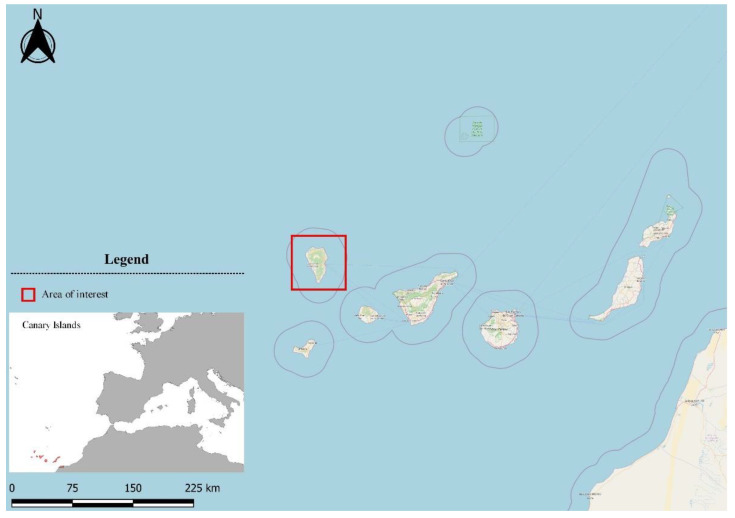
Location of La Palma island within the Canary archipelago.

The Canary Archipelago comprises a chain of seven major volcanic islands (Figure 1), which are located approximately 100 km off the north-west coast of Africa and are emplaced beneath oceanic crust, as evidenced by the identification of gabbro xenoliths with tholeiitic basalt-type composition [34,35,36]. This volcanic province is formed by a mantle plume subducting beneath a slowly moving oceanic lithosphere, evolving from NE to SW [37,38,39,40]. The magmatic activity, within the province started over 20 million years ago, when the eastern islands of Lanzarote and Fuerteventura began to develop. La Palma and Tenerife are currently the youngest and the most volcanically active islands, representing the present-day location of hotspot magmatism [41,42]. 

La Palma is the northwestern island of the Canary archipelago (Figure 1), covering an area of 708 km^2^. The very low precipitation and the relief of the island have resulted in the absence of a drainage network. The average annual temperature is close to 20 degrees Celsius, and the maximum altitude reaches 2426 m. The population of La Palma is 87 thousand, and the main economic activities are tourism and agriculture. According to a recent study [43], there are five main land use–land cover classes in the Canary Islands: forests and semi-natural areas, agricultural areas, artificial surfaces, water bodies and wetlands. In recent decades (1990–2018), an increase in artificial surfaces with a corresponding decrease in agricultural areas has been observed.

The geological structure of La Palma was described in detail in [38,42,44], while the precise aging of the formations was described by [45,46]. La Palma’s geological setting can be distinguished into three major units: the oldest one is the Basal Complex (c. 40 to 30 Ma), which consists of a Pliocene seamount sequence and a plutonic complex, which was uplifted and tilted by coeval intrusions [47]; the intermediate volcanic series (17 Ma to 400 ka); and the younger volcanic series (125 ka to present). The latter, named Cumbre Vieja, consists of numerous volcanic cones formed by lava and scoria, and occupies the southern part of the island. It is a broad lava field emplaced during the 1677–1678 eruption. Lava flows also reached the sea in 1585, 1646, 1712, 1949, 1971, and 2021.

On 19 September 2021, an eruption took place at the Cumbre Vieja volcanic ridge, revealing that the area is still active. Volcanic activity lasted from 19 September to 13 December 2021, while the eruption was officially declared to have ended on 25 December 2021—after 12 days without any activity. Therefore, this specific eruption is characterized as the longest and most damaging recorded volcanic eruption on La Palma. During the 85 days of volcanic activity, an area of 10 km^2^ was covered by lava, 3000 buildings were destroyed, and 7000 people were evacuated. According to the government of the Canary Islands, the total damage was estimated at about EUR 843 million.

### 2.2. SAR Data

The Sentinel-1 constellation constitutes the first mission of the European Union’s Earth observation program, named Copernicus. The constellation consists of two radar satellites (i.e., Sentinel-1A, Sentinel-1B) orbiting the same polar orbital plane. Sentinel-1A was launched in 2014, while the identical Sentinel-1B was placed into orbit in 2016. Both carry C-band SAR instruments (5.6 cm wavelength), operating at a center frequency of 5.405 GHz and providing data in single or dual polarization. The Sentinel-1 satellites are able to capture Earth images in four different acquisition modes (Stripmap, Interferometric Wide swath, Extra Wide swath, Wave) with spatial resolution varying from 5 to 40 m with coverage up to 400 km. The short revisit time and the free availability of the data are the main advantages of the mission.

**Figure 2:**
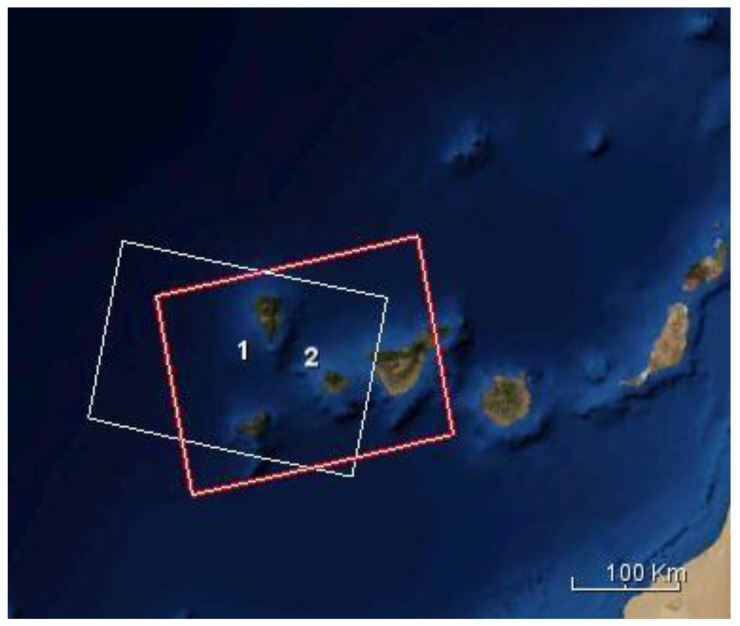
Sentinel-1 data frames. The red square corresponds to the Sentinel-1 data obtained during the ascending pass, while the white square represents the data frame for Sentinel-1 data collected during the descending pass.

The mapping of the ground deformation, caused by the volcanic eruption on La Palma island, was carried out using Sentinel-1’s interferometric wide swath mode imagery. This mode is the most suitable for land applications, allowing the scanning of a swath with a width of 250 km at a moderate spatial resolution (5 × 20 m). The homogeneous image quality over the entire surface of swath is ensured by Terrain Observation with Progressive Scans SAR (TOPSAR) technique, in which the radar’s beam is steered electronically from backward to forward in the azimuth direction for each burst while also steering the beam within the range. In the current study, Sentinel-1 IW Single Look Complex (SLC) and Ground Range Detected (GRD) data, acquired during the ascending and descending pass, were collected over La Palma island (Figure 2), throughout the eruptive phase (Table 1). The SLC products consist of focused and georeferenced SAR data, and include a single look in each dimension while maintaining phase information. In contrast, the GRD products consist of focused SAR data without phase information that have been detected, multilooked and projected on the basis of an ellipsoid model of the Earth.

### 2.3. Methods Applied

**Figure 3:**
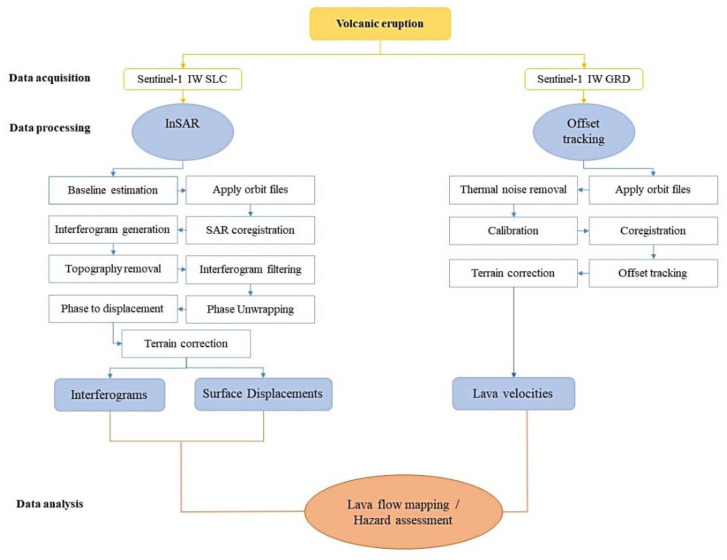
Flowchart describing the applied methodology.

The mapping of the surface deformation caused by the recent volcanic eruption on the island of La Palma was based on the processing of Sentinel-1 imagery via InSAR and offset tracking techniques. A flowchart presenting the main steps of the applied methodology is presented in Figure 3.

InSAR is a valuable technique that has been applied for the investigation of ground deformation emerging from geological, volcanic, tectonic, seismic or anthropogenic processes since 1992, in which year ERS-1 data were successfully used to capture the surface deformation caused by the Landers earthquake in Southern California [48,49,50,51,52]. Since then, the rapid advances in technology and the launch of new SAR satellites have contributed to more accurate measurements of surface deformation, and therefore a more comprehensive understanding of ongoing Earth processes. In this context, Sentinel-1 IW SLC products were obtained in order to map the surface deformation over La Palma. Processing was performed using the Sentinel Application Platform (SNAP) software, following a standard interferometric procedure. More specifically, the collected imagery was imported into the software, and critical interferometric parameters, including perpendicular baseline and coherence, were estimated for each Sentinel-1 interferometric pair (Table 2). The perpendicular baseline constitutes the projection of the interferometric baseline perpendicular to the slant range, while interferometric coherence is a correlation coefficient between the multitemporal SAR images. These two parameters can be used to determine whether interferometric pairs are suitable for further processing, or if they are dominated by phase noise. Subsequently, the data were corrected using accurate orbital information, and then SAR coregistration was applied. The next steps of InSAR processing consisted of the following procedures: interferogram generation, topography removal, interferogram filtering, phase unwrapping, phase to displacement conversion, and terrain correction (Figure 3). It is worth mentioning that the removal of the topographic phase component was carried out using a Shuttle Radar Topography Mission (SRTM) 1 Arc-Second Global DEM [53], while a Goldstein filter was applied as a phase filtering operation [54]. Monthly Sentinel-1 interferograms and the corresponding Line Of Sight (LOS) displacements were extracted as the end products of the procedure.

Despite the wide acceptance of InSAR techniques for post-eruption volcanic assessment [55,56], it was observed that phase-based deformation methods face some limitations in the monitoring of the evolution of the eruption associated with the loss of SAR coherence due to the significant changes in the terrain. The current research examined whether the amplitude-based methods for surface change identification could be a complementary source of information in the middle of volcanic emergencies. Thus, the Sentinel-1 IW GRD products were imported into SNAP, and they were processed using the offset tracking technique in order to estimate lava flow velocities on La Palma (Table 3). Specifically, the processing of the Sentinel-1 IW GRD products using the aforementioned approach consisted of: correction of the SAR imagery with the respective precise orbital data, the removal of thermal noise, radiometric calibration, co-registration, patch cross-correlation optimization (i.e., offset tracking), and terrain correction. The lava flow velocities were the final output of the procedure. The processing parameters of the applied approach were defined as described in [57].

## 3. Results

### 3.1. SAR Interferometry

**Figure 4:**
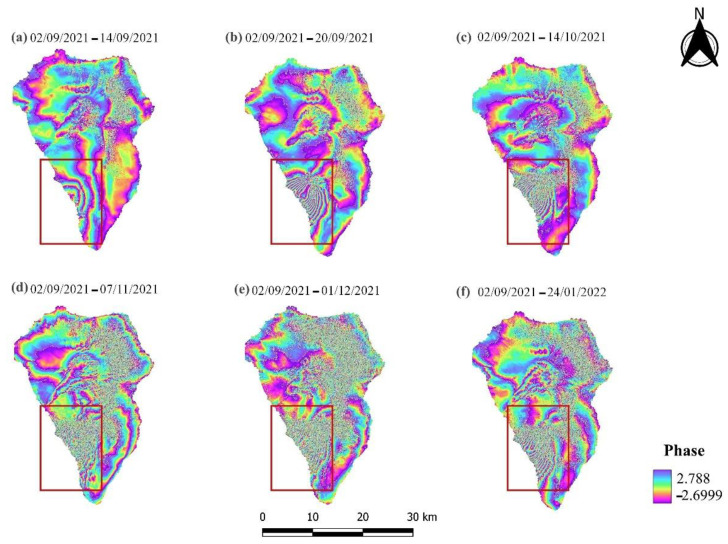
Multitemporal Sentinel-1 ascending interferograms. The red square indicates the area displaying the greatest deformation.

**Figure 5:**
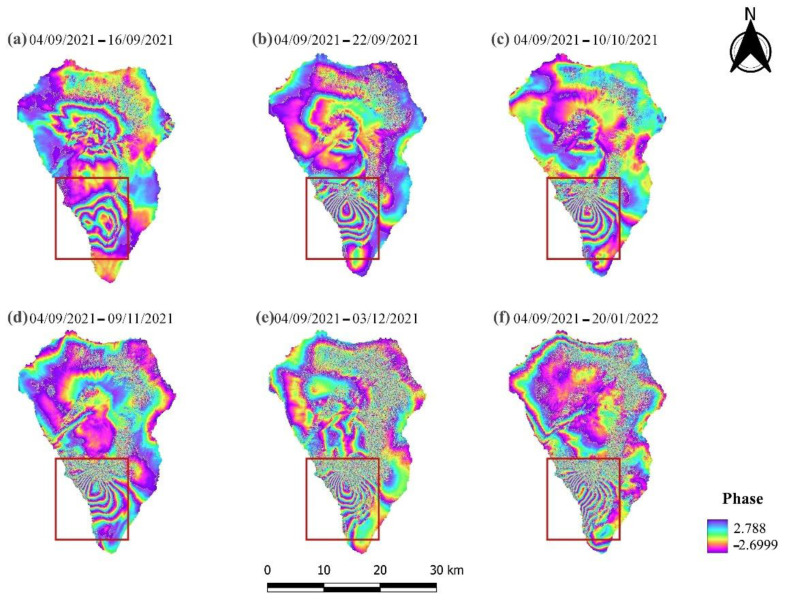
Multitemporal Sentinel-1 descending interferograms. The red square indicates the area displaying the greatest deformation.

The Sentinel-1 IW SLC products were processed in order to obtain multitemporal (one per month) interferograms (Figure 4 and Figure 5). In particular, the interferometric fringes, detected in the southwestern part of the island a few days before the eruption (19 September 2021) indicate that the area was deforming (Figure 4a and Figure 5a). The specific deformation is related to the upwelling of magma and ground inflation [58]. Meanwhile, dense interferometric fringes were observed in the same location in the ascending and descending interferograms, revealing the deformation caused to the island during the eruptive phase (Figure 4b–f and Figure 5b–f). The Sentinel-1 interferograms derived by processing the descending data were more suitable for deformation analysis, since they included cleaner and sharper interferometric fringes. Conversely, the interferograms created using Sentinel-1 ascending imagery were characterized by phase decorrelation, which is probably associated with the lower coherence values between the SAR acquisitions (Table 2). Moreover, it is obvious that with increasing temporal baseline of the selected interferometric pairs, there is a decrease in the quality of the generated interferograms.

**Figure 6:**
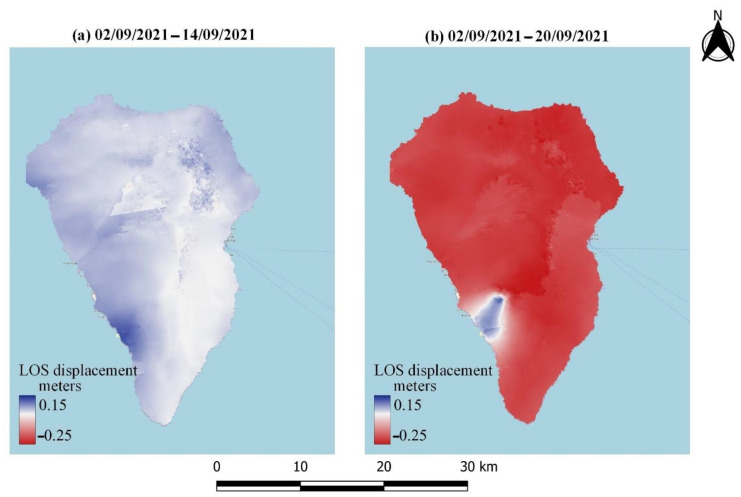
LOS displacement obtained by processing the Sentinel-1 ascending imagery. (**a**) LOS displacement before the eruption. (**b**) LOS displacement after the eruption.

**Figure 7:**
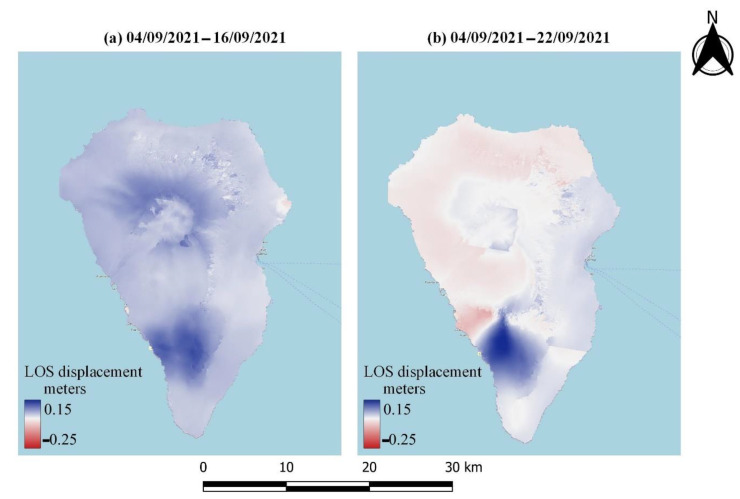
LOS displacement obtained by processing the Sentinel-1 descending imagery. (**a**) LOS displacement before the eruption. (**b**) LOS displacement after the eruption.

**Figure 8:**
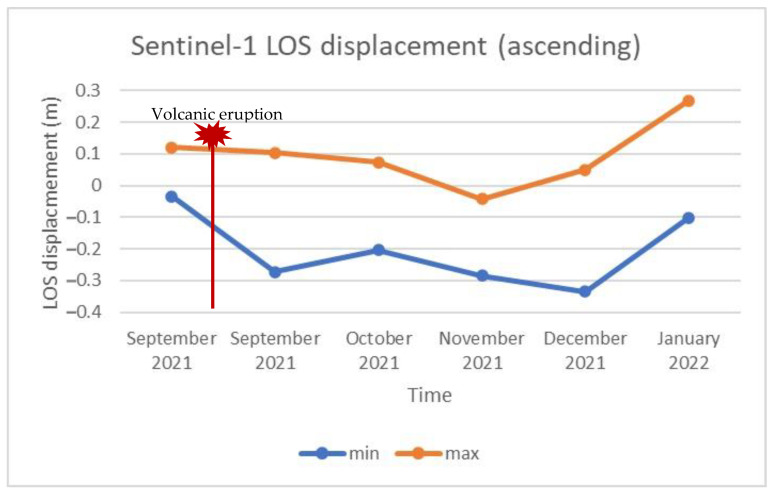
Multitemporal LOS displacement over the entire eruptive period, obtained by processing the Sentinel-1 ascending imagery.

**Figure 9:**
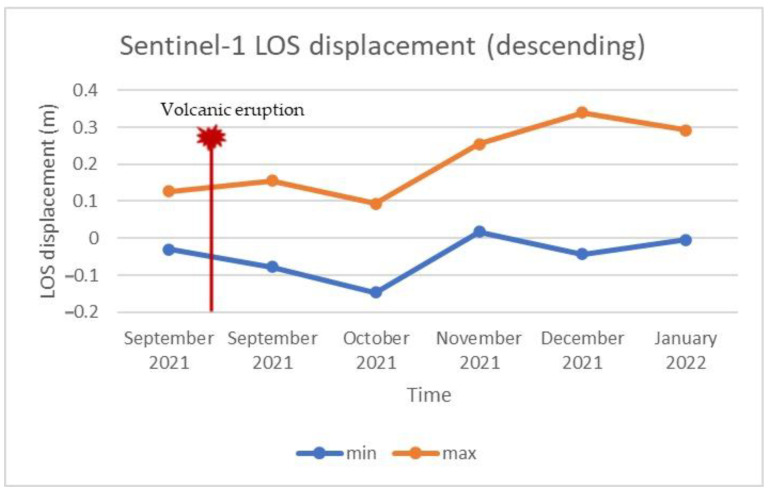
Multitemporal LOS displacement over the entire eruptive period, obtained by processing the Sentinel-1 descending imagery.

As already mentioned, LOS displacements were estimated by processing the acquired Sentinel-1 data. Figure 6 and Figure 7 depict the LOS displacements obtained by processing the data collected during ascending and descending pass, respectively, during different stages of volcanic activity. Specifically, Figure 6a and Figure 7a display the LOS displacements over the island before the volcanic eruption, while Figure 6b and Figure 7b present the corresponding LOS displacements after the eruption. A relative movement towards the satellite can be observed in blue color in Figure 6a and Figure 7a, suggesting ground inflation prior to the upcoming eruption [58]. Meanwhile, areas moving away from the satellite can be identified in both the ascending and descending data after the volcanic eruption (Figure 6b and Figure 7b). These areas are mapped in red, and are linked to the volcano’s deflation. The multitemporal evolution of the LOS displacements throughout the entire eruption period (i.e., from a few days before the eruption until the end of the eruptive phase) is depicted in Figure 8 and Figure 9, corresponding to ascending or descending imagery, respectively. Repeated episodes of subsidence and uplifts can be observed in both figures, and are closely associated with the various eruptive phases (i.e., inflation–deflation, crystallization and cooling, etc.).

### 3.2. Offset Tracking

**Figure 10:**
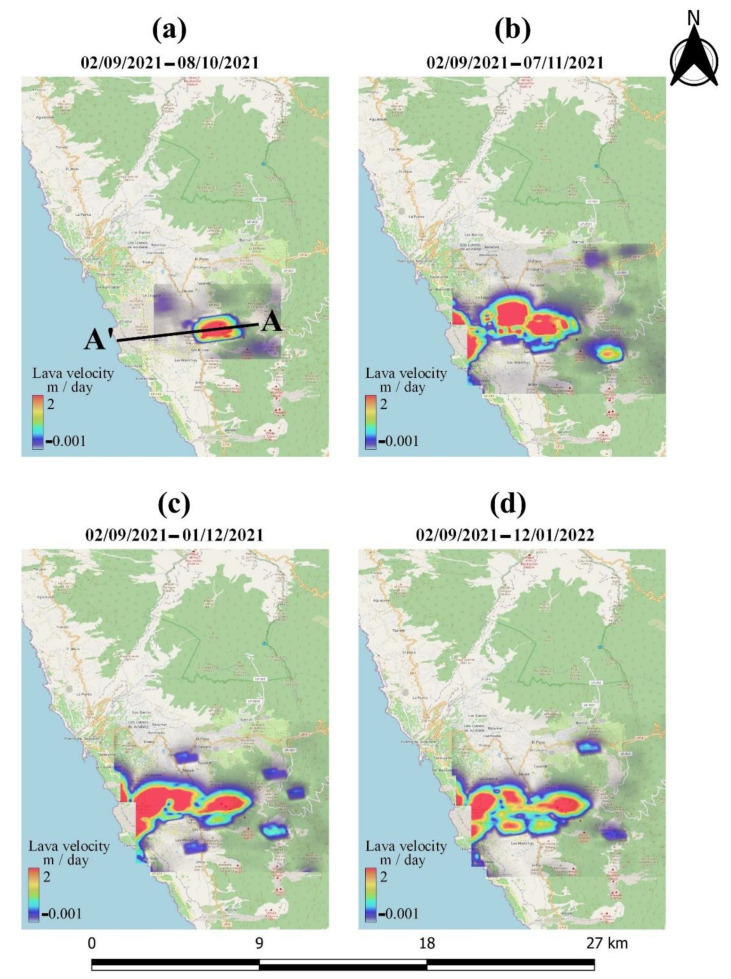
Lava flow evolution, obtained by processing the Sentinel-1 ascending data through offset tracking.

**Figure 11:**
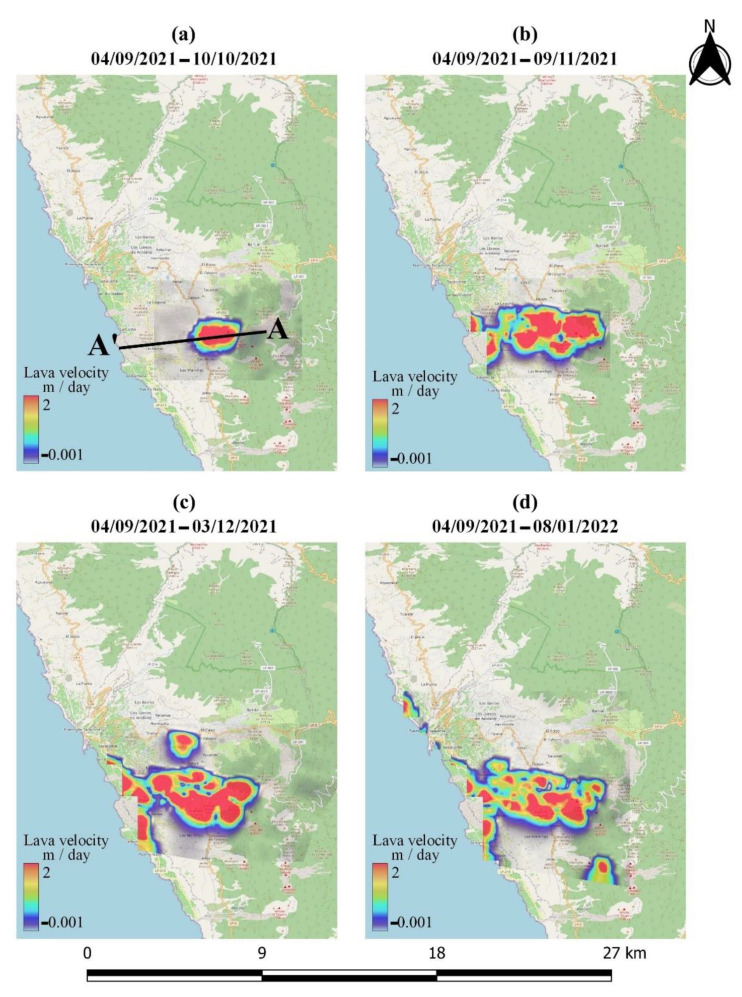
Lava flow evolution, obtained by processing the Sentinel-1 descending data through offset tracking.

**Figure 12:**
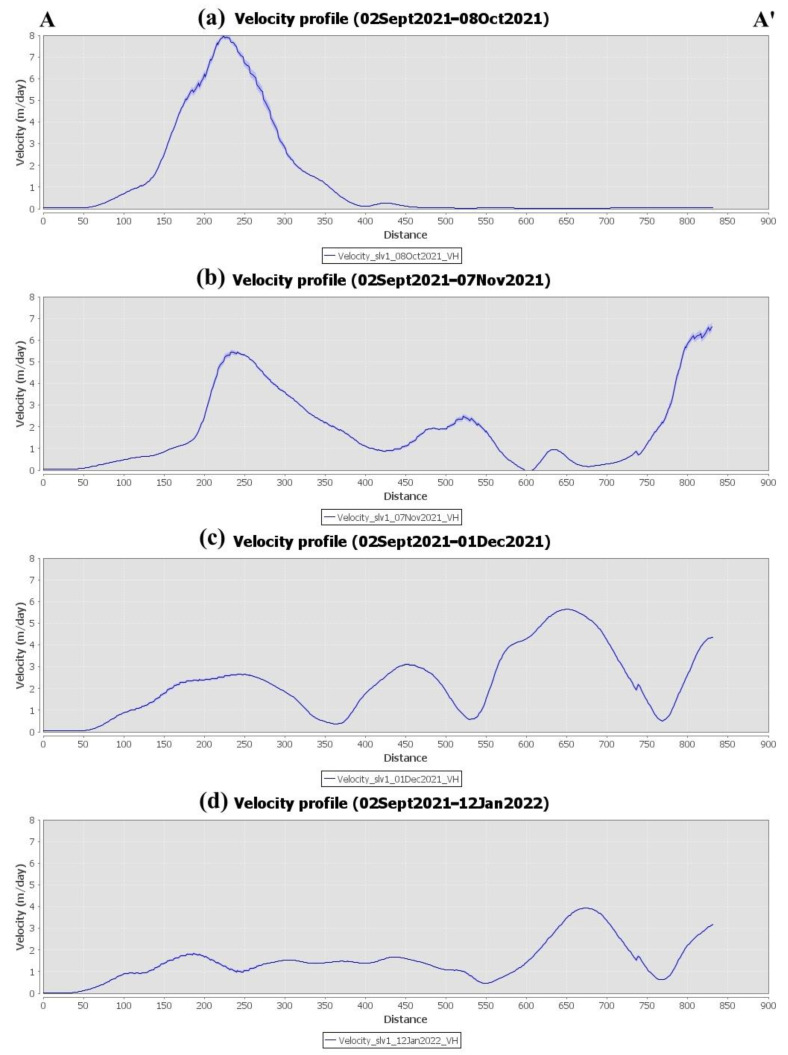
Lava flow velocity profiles of section AA’ throughout the different eruptive phases, obtained by processing the Sentinel-1 ascending data through offset tracking.

**Figure 13:**
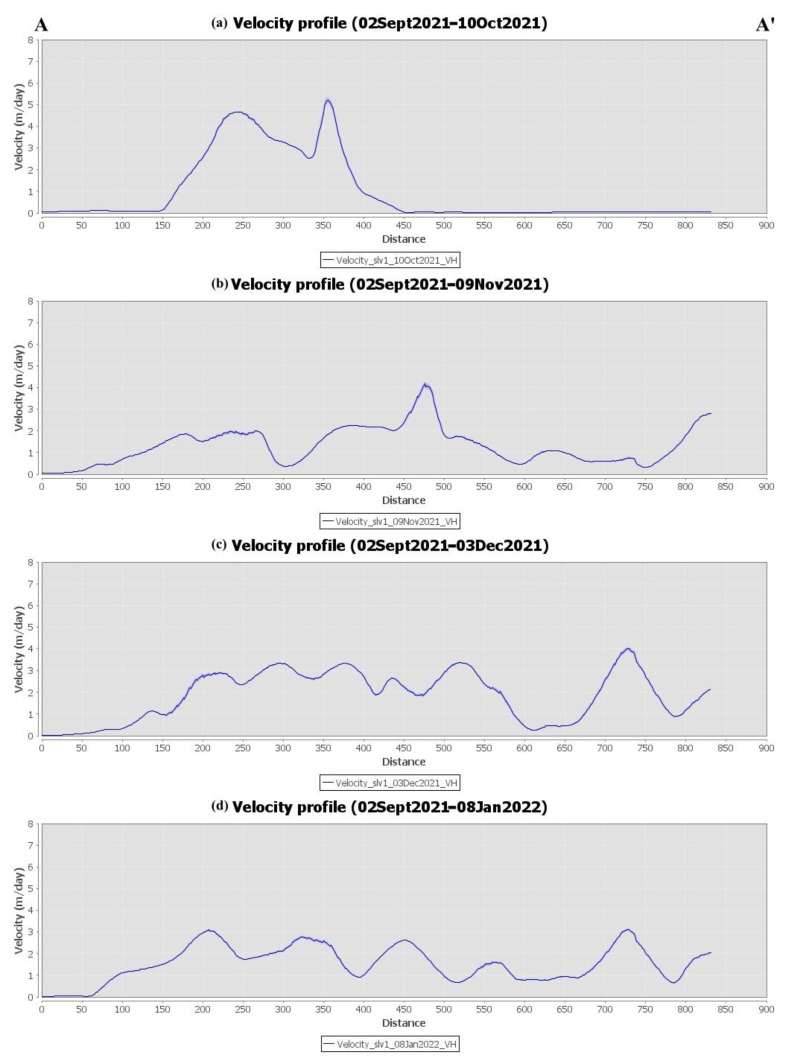
Lava flow velocity profiles of section AA’ throughout the different eruptive phases, obtained by processing the Sentinel-1 descending data through offset tracking.

Offset monitoring was used to monitor the evolution of lava flows on the island throughout the entire eruptive phase. This approach was used as a complementary source of information that cannot be extracted using conventional InSAR methods due to limitations related to coherence loss. The extent and velocity of lava flow during the various phases of the eruption, derived by processing the Sentinel-1 GRD ascending and descending products, are depicted in Figure 10 and Figure 11, respectively. High lava velocities (>2 m/day) are displayed in red, while blue corresponds to the lowest velocities. As can be observed in Figure 10a and Figure 11a, lava flow initially developed around the vents and then progressed westward down the slope, toward the coastline (Figure 10b and Figure 11b). The extent of the lava flow continued to expand, forming a lava delta (Figure 10c and Figure 11c), while lava flow velocity decreased dramatically after December (Figure 10d and Figure 11d). Section AA’ was defined (Figure 10a and Figure 11a) with the aim of performing a more detailed analysis of the evolution of lava flow velocity over time. Thus, lava flow velocity profiles were generated, either from ascending or descending Sentinel-1 data, during the various eruptive phases (Figure 12 and Figure 13). The highest lava flow velocities were recorded during the first eruptive phase, and ranged between 6 and 8 m/day (Figure 12a and Figure 13a).

## 4. Discussion

The immediate and accurate provision of operational information concerning volcanic activity—surface deformation as well as the spread of lava—are a critical and high-priority issue for communities living near volcanoes. In this context, the current study focused on analyzing the surface deformation occurring on the Cumbre Vieja volcanic ridge by means of InSAR and offset tracking. The operational capabilities of SAR (i.e., collection of data independent of weather conditions) have already been demonstrated in more than 500 volcanic monitoring case studies [59,60].

The latest La Palma eruption has been a research topic of interest for several scientists. In particular, recent studies have focused on the near-real-time mapping of active lava flows in subaerial hotspot eruptions using data provided by Fire Information for Resource Management System (FIRMS) [61], as well as in the analysis of the post-eruption topographic changes though the creation of high-resolution Digital Surface Models, obtained by Unmanned Aerial Vehicles [62,63]. Other researchers have attempted to create a numerical model to simulate the propagation of a tsunami that could potentially be caused by a submarine slide of the accumulated lava, placed on the island nearshore [64]. In addition, satellite remote sensing data have been used to identify the spatial extent and intensity of the impact of sulfur emissions in the pine forest of the island [65].

In terms of our research, the interpolation of the multitemporal interferograms, as well as the pre- and post-eruption LOS displacements, revealed that: (a) the island was uplifted before the eruption due to magma influx; and (b) the eruption was followed by a relatively rapid subsidence, arising from the removal of magma and gas. The detection of uplift in InSAR data prior to a potential future eruption has been observed and described in other cases [66,67]. Conversely, it has been demonstrated that the decrease in pressure in the magma reservoir after the eruption is reflected by an increase in subsidence in LOS displacements [67,68]. In fact, the aforementioned observation pattern, consisting of uplift and subsidence, is an important indication that a volcano is entering either an unrest period [69,70] or a post-unrest period [22]. Other researchers have related uplift and subsidence, as depicted in InSAR observations, to the morphology, geometry, and magma composition of the volcano under investigation [71].

**Figure 14:**
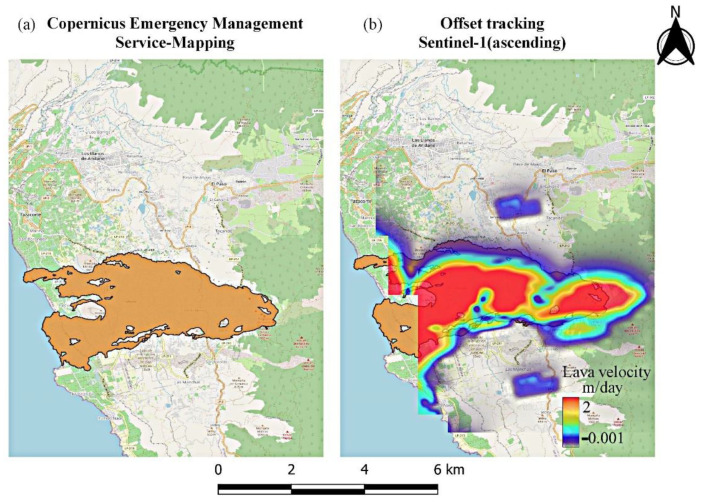
(**a**) Extent of lava flow, as released by Copernicus Emergency Management Service-Mapping [63]. (**b**) Lava flow, obtained by processing the Sentinel-1 ascending data through offset tracking.

As mentioned previously, we attempted to overcome the limitations of InSAR with respect to surface deformation monitoring related to the spread of lava flows by using the offset tracking technique. The results demonstrated that the approach was able to monitor the evolution of lava flows on the island throughout the entire eruptive period. Indeed, offset tracking outcomes are comparable to the lava flow area (Figure 14), derived from Copernicus Emergency Management Service-Mapping [72]. Although offset tracking is typically less accurate (~1/10 of pixel resolution), this approach can make an effective contribution to the measurement of large-scale surface displacements in both azimuth and slant range direction [73]. The main asset of the current study is the proposition of the offset tracking technique as a rapid and functional solution for the mapping of lava flows during a volcano’s eruptive phase. The existence of a variety of SAR satellites (Sentinel-1, Cosmoskymed, TerraSAR-X, ALOS, etc.) guarantees the acquisition of images during a volcanic crisis. Thus, SAR data are the only near-real-time source of information that can be used as an immediate solution for risk reduction and hazard management. In addition, the offset tracking processing time of a few hours (depending on network and computer capacities) makes it possible to achieve near-real-time computation of lava velocity. It is worth mentioning the amplitude-based change detection methods have been exploited to: (a) the monitoring, analysis and quantification of dome growth mechanisms and processes [30,74] and (b) the assessment of instabilities on volcano’s flanks [29]. As the approach proved quite promising, further investigation is required to enhance its robustness. In this context, the integration of higher-precision topographic information (Digital Elevation Models), could potentially yield better accuracy and spatial coverage of the extent of lava flow, thus improving monitoring ability.

## 5. Conclusions

The current study focused on mapping the ground deformation caused by the recent volcanic activity on the island of La Palma. In this context, Sentinel-1 products were collected and processed using InSAR and offset tracking techniques.

The key points of the research can be summarized as follows:

- Sentinel-1 interferograms are able to capture ground deformation related to either (a) the upwelling of magma and ground inflation a few days before the eruption, or (b) the eruptive phase.
- The relative movement towards the satellite indicated ground inflation prior to the upcoming eruption, while post-eruption deflation was accompanied by a relative movement away from the satellite.
- The multitemporal evolution of LOS displacements throughout the entire eruptive period was linked to the various eruptive phases (i.e., inflation–deflation, crystallization and cooling, etc.).
- Offset tracking was successfully used to monitor the evolution of lava flows on the island throughout the entire eruptive period.
- High lava velocities (>2 m/day) were detected.
- The analysis of the evolution of lava flows contributed to achieving a better understanding of the volcanic processes.

## Figures and Tables

**Table 1:** Sentinel-1 data.

a/a ^1^	Date ^2^	Mode ^3^	Pass ^4^	Track ^5^
1	02/09/2021	SLC	ascending	60
2	14/09/2021	SLC	ascending	60
3	20/09/2021	SLC	ascending	60
4	14/10/2021	SLC	ascending	60
5	07/11/2021	SLC	ascending	60
6	01/12/2021	SLC	ascending	60
7	24/01/2022	SLC	ascending	60
8	04/09/2021	SLC	descending	169
9	16/09/2021	SLC	descending	169
10	22/09/2021	SLC	descending	169
11	10/10/2021	SLC	descending	169
12	09/11/2021	SLC	descending	169
13	03/12/2021	SLC	descending	169
14	20/01/2022	SLC	descending	169
15	02/09/2021	GRD	ascending	60
16	08/10/2021	GRD	ascending	60
17	07/11/2021	GRD	ascending	60
18	01/12/2021	GRD	ascending	60
19	12/01/2022	GRD	ascending	60
20	04/09/2021	GRD	descending	169
21	10/10/2021	GRD	descending	169
22	09/11/2021	GRD	descending	169
23	03/12/2021	GRD	descending	169
24	08/01/2022	GRD	descending	169

^1^ Number of Sentinel-1 acquisitions. ^2^ Date of Sentinel-1 acquisition. ^3^ Sentinel-1 product type. ^4^ Orbital path of the mission. Ascending: the satellite moves northwards across the Earth. Descending: the satellite moves southwards across the Earth. ^5^ Relative orbit of Sentinel-1 acquisitions.

**Table 2:** Parameters of Sentinel-1 interferometric pairs (acquisition dates, product type, orbital path, baseline and coherence).

a/a	Interferometric Pair		Mode	Pass	Track	Baseline (m)	Coherence
1	02/09/2021	14/09/2021	SLC	ascending	60	−49.59	0.95
2	02/09/2021	20/09/2021	SLC	ascending	60	47.90	0.94
3	02/09/2021	14/10/2021	SLC	ascending	60	−41.53	0.93
4	02/09/2021	07/11/2021	SLC	ascending	60	3.57	0.94
5	02/09/2021	01/12/2021	SLC	ascending	60	94.97	0.84
6	02/09/2021	24/01/2022	SLC	ascending	60	22.88	0.85
7	04/09/2021	16/09/2021	SLC	descending	169	5.85	0.98
8	04/09/2021	22/09/2021	SLC	descending	169	−9.06	0.98
9	04/09/2021	10/10/2021	SLC	descending	169	−36.00	0.95
10	04/09/2021	09/11/2021	SLC	descending	169	27.08	0.92
11	04/09/2021	03/12/2021	SLC	descending	169	47.75	0.88
12	04/09/2021	20/01/2022	SLC	descending	169	28.98	0.85

**Table 3:** Parameters of Sentinel-1 pairs, as processed using offset tracking (acquisition dates, product type and orbital path).

a/a	Offset Tracking Pairs		Mode	Pass	Track
1	02/09/2021	08/10/2021	GRD	ascending	60
2	02/09/2021	07/11/2021	GRD	ascending	60
3	02/09/2021	01/12/2021	GRD	ascending	60
4	02/09/2021	12/01/2022	GRD	ascending	60
5	04/09/2021	10/10/2021	GRD	descending	169
6	04/09/2021	09/11/2021	GRD	descending	169
7	04/09/2021	03/12/2021	GRD	descending	169
8	04/09/2021	08/01/2022	GRD	descending	169

## Data Availability

The data presented in this study are available on request from the corresponding author. The data are not publicly available, due to privacy considerations.
